# Therapeutic Effects of *Heterotrigona itama* (Stingless Bee) Bee Bread in Improving Hepatic Lipid Metabolism through the Activation of the Keap1/Nrf2 Signaling Pathway in an Obese Rat Model

**DOI:** 10.3390/antiox11112190

**Published:** 2022-11-05

**Authors:** Zaida Zakaria, Zaidatul Akmal Othman, Joseph Bagi Suleiman, Khairul Mohd Fadzli Mustaffa, Nur Asyilla Che Jalil, Wan Syaheedah Wan Ghazali, Ninie Nadia Zulkipli, Mahaneem Mohamed, Khaidatul Akmar Kamaruzaman

**Affiliations:** 1 Department of Physiology, School of Medical Sciences, Universiti Sains Malaysia, Kubang Kerian 16150, Kelantan, Malaysia; 2 Unit of Physiology, Universiti Sultan Zainal Abidin, Kuala Terengganu 20400, Terengganu, Malaysia; 3 Department of Science Laboratory Technology, Akanu Ibiam Federal Polytechnic, Unwana P.O. Box 1007, Ebonyi State, Nigeria; 4 Institute for Research in Molecular Medicine, Universiti Sains Malaysia, Kubang Kerian 16150, Kelantan, Malaysia; 5 Department of Pathology, School of Medical Sciences, Universiti Sains Malaysia, Kubang Kerian 16150, Kelantan, Malaysia; 6 Unit of Integrative Medicine, School of Medical Sciences, Universiti Sains Malaysia, Kubang Kerian 16150, Kelantan, Malaysia

**Keywords:** *Heterotrigona itama*, bee bread, high-fat diet, obesity, MAFLD, oxidative stress, lipid metabolism, Keap1/Nrf2 pathway, SIRT1, AMPK

## Abstract

Bee bread (BB) has traditionally been used as a dietary supplement to treat liver problems. This study evaluated the therapeutic effects of *Heterotrigona itama* BB from Malaysia on obesity-induced hepatic lipid metabolism disorder via the regulation of the Keap1/Nrf2 pathway. Male *Sprague Dawley* rats were fed with either a normal diet or high-fat diet (HFD) for 6 weeks to induce obesity. Following 6 weeks, obese rats were treated either with distilled water (OB group), BB (0.5 g/kg body weight/day) (OB + BB group) or orlistat (10 mg/kg body weight/day) (OB + OR group) concurrent with HFD for another 6 weeks. BB treatment suppressed Keap1 and promoted Nrf2 cytoplasmic and nuclear translocations, leading to a reduction in oxidative stress, and promoted antioxidant enzyme activities in the liver. Furthermore, BB down-regulated lipid synthesis and its regulator levels (SIRT1, AMPK), and up-regulated fatty acid β-oxidation in the liver of obese rats, being consistent with alleviated lipid levels, improved hepatic histopathological changes (steatosis, hepatocellular hypertrophy, inflammation and glycogen expression) and prevented progression to non-alcoholic steatohepatitis. These results showed the therapeutic potentials of *H. itama* BB against oxidative stress and improved lipid metabolism in the liver of obese rats possibly by targeting the Keap1/Nrf2 pathway, hence proposing its role as a natural supplement capable of treating obesity-induced fatty liver disease.

## 1. Introduction

The worldwide prevalence of overweight and obesity has increased by two-folds since 1980 to an extent that almost a third of the global population is now considered to be overweight or obese [1]. Obesity adversely affects most of the body’s physiological functions and is regarded as “the biggest public health threat for this century”. It is caused by various factors, including a high-fat diet (HFD), and increases the risk of developing numerous co-morbidities, including type 2 diabetes mellitus, insulin resistance, hyperlipidemia, several types of cancers, cardiovascular diseases and liver diseases [2].

Metabolic dysfunction-associated fatty liver disease (MAFLD), previously known as non-alcoholic fatty liver disease (NAFLD) [3,4] is strongly related to obesity and is recognized as a complex metabolic syndrome of abnormal liver metabolism. There is growing evidence that supports a key role of oxidative stress resulting from the generation of reactive oxygen species (ROS) in the progression of MAFLD and its progressive form non-alcoholic steatohepatitis (NASH) [5]. Mitochondria are the primary intracellular sites of oxygen consumption; hence, they are the main source of ROS generation in MAFLD. It is believed that excessive hepatic lipid buildup leads to structural abnormalities of hepatic mitochondria and impairment of the electron chain, as well as augmented lipid peroxidation and ROS generation, which further stimulates oxidative stress and inflammation in the liver [6,7,8]. As oxidative stress is classified as the disequilibrium between ROS and antioxidants, a counterbalance by a complex antioxidant defense system such as the key antioxidant enzymes superoxide dismutase (SOD), catalase (CAT), glutathione peroxidase (GPx), glutathione-S-transferase (GST) and glutathione reductase (GR) is necessary to avoid the generation of ROS and henceforth oxidative stress in the liver. Previous studies have demonstrated the correlation between the reduction in antioxidant enzyme activities and the severity of MAFLD in clinical and animal studies [9,10,11,12]. Additionally, it has also been reported that the activation of nuclear factor erythroid 2-related factor 2 (Nrf2), which is the key transcription factor regulating the expression of antioxidant enzyme genes [13], results in the inhibition of oxidative stress and a reduction in hepatic inflammation and fibrosis [14]. Under quiescent cellular environments, Nrf2 remains inactive by forming a complex with Kelch-like ECH-associated protein 1 (Keap1), a Nrf2 repressor protein in the cytoplasm. However, during exposure to oxidative stress and electrophilic substances, Keap1 becomes oxidized and releases Nrf2 to be translocated to the nucleus where it stimulates the transcription of the gene containing the antioxidant response element (ARE) which activates the translation of antioxidant genes [15]. Numerous studies have indicated the effects of sterol regulatory element binding protein-1c (SREBP-1c) or peroxisome proliferator-activated receptor alpha (PPARα)-dependent pathways on lipogenesis and fatty acid oxidation, respectively, in hepatic lipid metabolism [16]. SREBP-1c controls the activities of lipogenic enzymes including fatty acid synthase (FAS) and its expression is up-regulated by the increased insulin level in the circulation and liver [17]. Meanwhile, PPARα has been demonstrated to be involved in the oxidation of fatty acids and mediates the activity of carnitine palmitoyltransferase 1α (CPT1α), which catalyzes the rate-limiting step in fatty acid β-oxidation. Consequently, a reduced level of PPARα leads to hyperlipidemia and lipid deposition in the hepatocytes [18]. In addition, both AMPK-activated protein kinase (AMPK) and sirtuin 1 (SIRT1) signaling also play a role in maintaining an energy balance and regulating lipid metabolism in the liver, muscle and adipose tissue [19,20]. Activated AMPK can suppress the levels of lipogenic-related protein including SREBP1 and FAS, as well as up-regulate the activities of enzymes responsible for fatty acid β-oxidation (PPARα and CPT1); hence, it can reduce lipid accumulation [21]. Likewise, an increased level of lipid accumulation in the liver of obese mice has been linked with the inhibition of AMPK and SIRT1 activities, hence aggravating the development of MAFLD [22]. Of note, it has also been reported that the activation of the Keap1/Nrf2 signaling pathway to maintain a redox status mediates lipid metabolism-related genes (SREBP-1c and PPARα) to reduce hepatic steatosis in an HFD-induced obese animal model [23]. Thus, the hepatotherapeutic agents with anti-oxidative stress may have a potential therapeutic modality on lipid accumulation.

Generally, stingless bees are found mainly in tropical and subtropical regions of the world, including Africa, Southeast Asia, Australia and South America. In Malaysia, over 30 species of stingless bee (*Trigona* spp.), which is also known as ‘kelulut’, have been documented in Peninsular Malaysia and out of this, 17 species were identified to inhabit the virgin forest [24]. The most common species of *Trigona* spp. in Malaysia is *Heterotrigona itama*, which is reared commercially due to high demand [25]. Bee bread is formed from the fermentation of mixtures of nectar, pollen and digestive enzymes secreted from the bee’s salivary glands [26,27]. The chemical compositions of bee bread are mainly comprised of proteins, free amino acids, carbohydrates, fatty acids, a wide range of sugars and high reducing sugars [28,29] and vitamins [30]. Moreover, each bee bread has a different composition which varies from different regions, climatic and seasonal variations, floral origins [31,32] and soil type [27]. Numerous data have shown that bee bread produced by *H. itama* possesses beneficial therapeutic effects on metabolic diseases including cardiovascular disease [33,34,35], renal disorder [36] and male infertility [37,38,39]. Moreover, our prior study revealed that concurrent administration of HFD and *H. itama* bee bread at 0.5 g/kg b.w./day for 12 weeks exhibited protection against obesity, liver oxidative damage and inflammation, as well as prevented the liver from NASH and fibrosis in rats [40]. Bee bread was also previously demonstrated to have an anti-lipogenic effect by reducing the expressions of FAS and acetyl-CoA carboxylase (ACC) in the obesity-induced fatty liver disease rat model [41]. Furthermore, numerous findings have been reported on the anti-obesity effects of flavonoids and phenolic acids, hence, increasing the liver antioxidant and detoxification system as well as improving lipid metabolism [42,43,44,45]. In this current study, we investigated whether the intake of *H. itama* bee bread would have beneficial therapeutic effects on liver oxidative stress and lipid accumulation after the induction of obesity in rats. Moreover, the underlying molecular mechanism by which bee bread alleviates redox imbalance and lipid metabolism disorder via the activation of the Keap1/Nrf2 pathway was explicated.

## 2. Materials and Methods

### 2.1. Bee Bread Collection and Preparation

Bee bread sample from the stingless bee *H. itama* was self-harvested and collected from a local stingless bee farm (Mentari Technobee PLT, Kelantan, Malaysia). The sample initial weight was measured, dried using a food dehydrator (Domani Industries Ltd., Foshan, China) (35 °C) and ground into powder form before being stored in −20 °C until further use [33,38].

### 2.2. High-Performance Liquid Chromatography (HPLC) Detection and Quantification of Polyphenolic Compounds

HPLC was performed based on the method published by Suleiman et al. (2021) [46]. Bee bread powder was suspended in water and methanol to produce aqueous and methanol solutions, respectively, to achieve final concentrations of 100 mg/mL. The solutions were then vortexed, sonicated and followed with centrifugation at 20,111× g for 5 min prior to HPLC analysis. The samples were analyzed using a Dionex RS3000 system (Thermo Scientific, Waltham, MA, USA). The chromatic separation was achieved at 25 °C on a Zorbax SB-C18 column (3.5 µm, 4.6 mm I.D × 150 mm) (Agilent Technologies, Santa Clara, CA, USA). A binary solvent system was employed consisting of 0.1% formic acid in water as solvent A and 0.1% formic acid in methanol (40:60, *v*/*v*) as solvent B. The chromatographic analyses were conducted at a run time of 0, 20, 25, 25.1 and 30 min. The flow rate was 1.0 mL/min, and the injection volume was 20 µL. The eluted components were monitored at 340 nm. The standard substances of gallic acid, caffeic acid, mangiferin, trans-ferulic acid, 2-hydroxycinnamic acid, trans-3-hydroxycinnamic acid, quercetin, kaempferol and apigenin were purchased from Sigma (St. Louis, MO, USA) and used as reference compounds.

### 2.3. Animals and Diet

Thirty-two male *Sprague Dawley* rats aged from 8 to 10 weeks (200–230 g) were purchased from the Laboratory Animal Research and Service Centre (ARASC), Universiti Sains Malaysia (USM). All the animal experiments complied with the National Institute of Health Guide for the Care and Use of Laboratory Animals and were approved by the USM Institutional Animal Care and Use Committee (IACUC) (USM/IACUC/2018/(113)(933)). Each rat was individually housed in a polypropylene cage with sterilized husk bedding in a room with a 12 h light–dark cycle, a controlled temperature at 22–24 °C and a relative humidity of 55–70%. All the rats had free access to a normal rat chow pellet and clean drinking water during one week of the acclimatization period. 

All animals were fed with either a normal diet or an HFD. The normal diet was a standard Altromin pellet (Altromin Spezialfutter GmbH & Co. KG, Lage, Germany) composed of soy, wheat and corn with approximately 24% protein, 64% carbohydrates and 12% fat in terms of caloric content. Meanwhile, the HFD was prepared based on a prior study with slight modifications, composed of 32 g of animal ghee, 68 g of powdered normal diet, 12% of cholesterol powder, as well as 300 mg calcium and 100 IU vitamin D3, which contained approximately 12% protein, 46% carbohydrate and 31% fat in terms of the caloric content [47].

### 2.4. Experimental Design

After adaptation, all the rats were indiscriminately divided into two experimental groups and fed ad libitum as follows: (1) Control (CON) group (*n* = 8); rats fed a normal diet and 1 mL distilled water once daily for 12 weeks, (2) HFD group (*n* = 24): rats fed HFD and oral gavage of 1 mL distilled water for 6 weeks to induce obesity. The Lee obesity index was used to confirm the obesity using a previously reported formula [48]: $\frac{\sqrt[{3}]{body}weight\;\left(g\right)}{naso-anal\;length\;\left(cm\right)}\times 1000$ and a value of Lee obesity of more than 315 was considered as obese [49]. Following 6 weeks, the confirmed obese rats in the HFD group were then separated into 3 random experimental groups (*n* = 8/group), and treated as follows for 6 weeks: (1) Obese group (OB): rats fed HFD and oral gavage of 1 mL distilled water, (2) Bee bread group (OB + BB): rats fed HFD and oral gavage of bee bread (0.5 g/kg b.w./day), and (3) Orlistat group (OB + OR): rats fed with HFD and oral gavage of orlistat (10 mg/kg b.w./day). A pilot study was conducted to identify the best dose of bee bread (0.5, 1.0 and 1.5 g/kg b.w./day) for improving some hepatic parameters in obese male rats. After 6 weeks, the findings demonstrated that the best dose of bee bread 0.5 g/kg b.w./day was selected for this animal study as this dose exerted the most improvement on the Lee obesity index, liver function and hepatic steatosis in obese male rats (Table S1 and Figure S1). Meanwhile, the dose of orlistat (Xepa-Soul Pattinson Sdn. Bhd. Melaka, Malaysia) at 10 mg/kg b.w./day was selected based on a prior reported study on obese rats [50]. Both bee bread and orlistat were weighed before being dissolved in distilled water to a final volume of 1 mL before being dispensed to the animals. 

### 2.5. Measurements of Obesity Parameters and Nutritional Composition

Body weights were monitored weekly throughout the experiment and the changes in the body weight between week 12 and week 0 were recorded as body weight gain. The Lee obesity index was calculated at the end of the experimental period. The food intake was monitored daily and the averages of food and calorie intakes for each rat were determined.

### 2.6. Blood and Tissue Collection

At the end of the 12th week, all the animals were anaesthetized intraperitoneally using 90 mg/kg ketamine and 5 mg/kg xylazine following a 12 h fast. Then, blood was collected from the rat’s posterior vena cava in tubes containing a gel clot activator. The blood samples were centrifuged (3000× g, 15 min) to obtain the serum and stored at −80 °C for biochemical assays. Meanwhile, the livers from each experimental group were dissected, rinsed in ice-cold normal saline solution, blotted dry and cut into three portions. The liver tissue was stored in the RNAlater (Sigma-Aldrich, St. Louis, MO, USA) at −80 °C for qRT-PCR analysis. The second portion of tissue was homogenized using a tissue homogenizer (IKA Labortechnik Co., Ltd., Wilmington, NC, USA) in 10 volumes of ice-cold phosphate buffer saline (pH 7.4) and centrifuged (3000× g, 20 min). The separated supernatant was stored at −80 °C until further use for liver biochemical analyses. The last portion of the liver tissue was rapidly fixed in 10% formalin for at least 48−72 h for immunohistochemical and histopathological analyses.

### 2.7. Determination of Serum Glucose, Insulin and HOMA-IR

Serum glucose (Qayee-Bio Life Science Co., Ltd., Shanghai, China) and insulin (Elabscience Biotechnology Inc. Co., Ltd. Wuhan, Hubei, China) levels were tested with commercially available kits according to the manufacturers’ instructions. The homeostatic model of assessment-insulin resistance (HOMA-IR) was calculated as referred to in the prior study [51].

### 2.8. Evaluations of Lipid Profiles

The levels of both triglyceride (TG) and total cholesterol (TC) in the serum were assessed using a commercialized kit (ARCHITECT c kit, Abbott, IL, USA) according to an enzymatic colorimetric method. Meanwhile, the level of low-density lipoprotein (LDL) was measured according to a formula as described in the previous study [52], i.e., LDL (mmol/L) = (TC − HDL − (TG/5). Furthermore, the level of high-density lipoprotein (HDL) was determined using Biosino Direct HDL-Cholesterol reagent kit (Biosino Bio-Technology and Science Inc., Beijing, China) by the eliminations of LDL-Cholesterol, chylomicron and VDLD-Cholesterol by cholesterol oxidase, cholesterol esterase and catalase.

### 2.9. Liver Biochemical Analyses

The hepatic TG, TC, SIRT1 and AMPK concentrations were determined using the commercial kits obtained from Qayee-Bio Life Science Co., Ltd. (Shanghai, China), referring to the manufacturers’ protocols. Meanwhile, nitric oxide (NO) concentration was assayed with common commercially available biochemical kits obtained from Elabscience Biotechnology Inc. Co., Ltd. (Wuhan, China) according to the manufacturer’s instruction. The level of lipid peroxidation in the liver was determined as a thiobarbituric acid reactive substance (TBARs) according to Chatterjee et al. (2000) [53]. The absorbance of a colored complex produced from the reaction of thiobarbituric acid and malondialdehyde was measured at 532 nm. The SOD activity was calculated according to a method by Al Batran et al. (2013) [54]. The enzyme activity was assessed by the measurement of diformazone, a final product formed from a reduced superoxide ion by tetrazolium blue nitro (NBT) at wavelength 560 nm. The CAT activity was evaluated according to Góth, (1991) [55] based on the enzyme-catalyzed decomposition of hydrogen peroxide and an assay of the remaining hydrogen peroxide with molybdate ions. The enzyme activity was assessed by measurement of the yellowish complex formed from the reaction spectrophotometrically (BioTek Instruments, Winooski, VT, USA) at 405 nm. The activity of GPx was assessed according to Doǧan et al. (1994) [56]. The enzyme activity was evaluated by the measurement of the change in the concentration of NADPH at wavelength 340 nm. Estimation of GST activity in the liver was measured according to Habig et al. (1974) [57] which was based on glutathione conjugation to 1-chloro-2,4-dinitrobenzene (CDNB) as a substrate. The enzyme activity was measured spectrophotometrically at wavelength 340 nm. The activity of GR was estimated according to Luchese et al. (2009) [58] based on the reduction of GSSG catalyzed by GR in the presence of NADPH to form GSH and NADP^+^. The decrease in absorbance due to the decreased concentration of NADPH was determined spectrophotometrically at 340 nm. The level of total glutathione (GSH) was evaluated according to Annuk et al. (2001) [59] with some modifications. The rate of yellow complex (5-thio-2-nitrobenzoic acid) formed from the reaction between the sulfhydryl group of GSH and 5,5′-dithiobis-2-nitrobenzoic acid (DTNB) was measured at wavelength 405 nm. The liver total antioxidant capacity (TAC) was evaluated by referring to a former described method by Koracevic et al. (2001) [60]. In this assay, the antioxidants from the liver homogenate inhibited the formation of TBARS and the reaction was calculated by measuring spectrophotometric absorbance at 532 nm. The protein contents of these samples were quantified using a bicinchoninic acid (BCA) protein assay kit according to the manufacturer’s instructions (Thermo Fisher Scientific, Inc., Waltham, MA, USA) and normalized to the data of liver tissue biochemical parameters. 

### 2.10. RNA Extraction and RT-qPCR Analysis

Total RNA from the liver tissue was extracted using Innu Prep RNA mini kit (Analytik Jena, Jena, Germany) and treated with PureLink^TM^ DNase (Invitrogen, Thermo Fisher Scientific Inc. USA) to remove any contaminating genomic DNA according to the manufacturers’ instructions. The concentration and purity of the RNA preparation were quantified by measuring the absorbances at 260 and 280 nm on a μDrop^TM^ Plate (Thermo Fisher Scientific Inc., Waltham, MA, USA) and only samples with OD260/280 of 1.8–2.0 were included in this study. Then, the quality and integrity of RNA were assessed using agarose gel electrophoresis stained with fluorescent dye in 1x LB buffer (Faster Better Media LLC, Hunt Valley, MD, USA). RT-qPCR was carried out in a 20 µL volume reaction containing 10 µL of SYBR Lo-ROX One-step Mix, 10 µM of each primer, 0.2 µL of reverse transcriptase, 0.4 µL of RNase inhibitor, 16 µL of DEPC-treated water and 4 µL of RNA (SensiFAST^TM^ SYBR Lo-ROX One-step kit, Bioline USA Inc., Taunton, MA, USA) using Stratagene Mx3000P qPCR system (Agilent Technologies, Santa Clara, CA, USA). Thermal cycling conditions included cDNA synthesis at 45 °C for 10 min, polymerase activation at 95 °C for 2 min, PCR amplification for 40 cycles each one consisting of denaturation at 95 °C for 5 s, annealing at 60 °C for 10 s and extension at 72 °C for 5 s, followed by melt curve stage. All primers were selected from GenBank and synthesized by Integrated DNA Technologies (IDT, Malaysia). The GAPDH gene was used as an internal control to normalize target gene expressions. Three replicates of each reaction were conducted and the relative mRNA transcription levels were calculated according to the method of the 2^−ΔΔCt^ method [61]. The primer sequences used for this study are shown in Table 1.

### 2.11. Immunohistochemical Detections of Keap1 and Nrf2 Expressions

Serial sections of formalin-fixed, paraffin-embedded liver samples were de-waxed, rehydrated and subjected to antigen retrieval treatment in a Tris-EDTA buffer solution (0.1 M, pH 9.0) with 0.05% Tween-20. The endogenous peroxide activity was blocked for 5 min using 3% hydrogen peroxide. Then, the liver sections were incubated using rabbit polyclonal primary antibodies against Keap1 and Nrf2 (Cloud- Clone Corp, Katy, TX, USA) (1:100) at 4 °C overnight. The primary antibodies were detected using a secondary antibody containing goat anti-rabbit (Dako EnVision^TM^ + System/HRP labelled polymer) (Agilent Technologies, Inc., Santa Clara, CA, USA). Immunospecific reactivity was visualized by Dako 3,3′-diaminobenzidine (DAB) chromogen substrate reagent (1:1) mixed solution (Agilent Technologies, Inc., Santa Clara, CA, USA), counterstained with hematoxylin (Merck, Darmstadt, Germany), then dehydrated in alcohol and xylene before being mounted. The protein expressions of Keap1 and Nrf2 were analyzed by two independent pathologists (blinded to the treatment the rats received) according to a previous study [64]. The immunoreactive score was assessed by multiplying the staining intensity (0, colorless; 1, light yellow; 2, brownish yellow; 3, brown) with a percentage of positively stained cells (0, negative; 1, 10%; 2, 11–50%; 3, 51–75%; 4, 75–100%). 

### 2.12. Liver Histopathological Examination

The formalin-fixed liver tissues were dehydrated in a series of ethanol and embedded in paraffin wax. Sections (3-µm thick) were cut and stained with hematoxylin-eosin (H&E) (both Merck, Darmstadt, Germany) and analyzed under an Olympus BX41 (Olympus Co., Tokyo, Japan). The histopathological changes such as the hepatocellular vesicular steatosis (i.e., macro- or micro-vesicular steatosis and hypertrophy) and inflammatory cell infiltration were assessed and followed by grading and scoring referring to the NAFLD activity score (NAS) [65,66] as follows: NASH; ≥5 and non-NASH; ≤3. A periodic acid-Sciff (PAS) staining was performed to detect glycogen accumulation (magenta color staining) in liver tissue. The glycogen score was evaluated following the percentage of positively stained cells as follows: 0 (0–15%), 1 (16–25%), 2 (26–50%) or 3 (76–100%) [67]. All the histopathological assessments were carried out by two liver pathologists blinded to the diet.

### 2.13. Statistical Analysis

Statistical analysis was carried out using GraphPad Prism, 8th Version Software (GraphPad Software Inc., Maryland, USA). All data were checked for normality and variance of the data sets using the Shapiro–Wilk and D’Agostino–Pearson Omnibus normality test, respectively. All data are expressed as means ± standard error of the means (SEM). One-way ANOVA was used followed by Tukey post-hoc test to determine the differences between the groups. *p* < 0.05 was considered to be statistically significant.

## 3. Results

### 3.1. Phenolic Compound Analysis of H. itama Bee Bread Using High-Performance Liquid Chromatography (HPLC)

**Figure 1:**
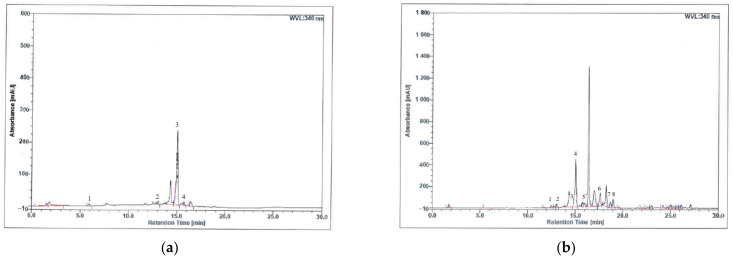
HPLC profiles of (**a**) aqueous and (**b**) methanol extracts of bee bread samples at 340 nm. (**a**) Peak 1, gallic acid; peak 2, mangiferin; peak 3, trans 3-hydroxycinnamic acid; peak 4, 2-hydroxycinnamic acid. (**b**) Peak 1, caffeic acid; peak 2, mangiferin; peak 3, trans ferulic acid; peak 4, trans 3-hydroxycinnamic acid; peak 5, 2-hydroxycinnamic acid; peak 6, quercetin; peak 7, kaempferol; peak 8, apigenin.

Figure 1 shows the HPLC profiles of (a) aqueous and (b) methanol bee bread extracts, respectively. The retention time point of the standards was compared to the HPLC profiles of both bee bread extracts, and the quantities for each of the chemical markers within the bee bread extracts were calculated and are summarized in Table 2. Aqueous bee bread extract had a relatively higher concentration of trans-3-hydroxycinnamic acid, followed by 2-hydroxycinnamic acid, gallic acid and mangiferin. Similarly, the methanol bee bread extract possessed a relatively higher concentration of trans-3-hydroxycinnamic acid, followed by quercetin, apigenin, kaempferol, 2-hydroxycinnamic acid, mangiferin, caffeic acid and trans-ferulic acid (Table 2).

### 3.2. Effects of H. itama Bee Bread on Obesity Parameters and Nutritional Composition

To determine the anti-obesity property of bee bread supplementation in obese rats, the Lee obesity index and body weight gain were recorded in the present study. As demonstrated in Table 3, 12-week HFD feeding significantly increased (*p* < 0.05) the Lee obesity index and body weight gain in the OB group. Meanwhile, the administration of bee bread significantly reduced (*p* < 0.05) these parameters. Similar patterns of results were also present in the OB + OR group.

Furthermore, no significant differences (*p* > 0.05) were found in the average food intake among all the experimental groups. However, all animals fed with the HFD demonstrated significant increases (*p* < 0.05) in the average of calorie intake compared to the CON group. Nonetheless, no significant differences (*p* > 0.05) were found in the average calorie intake between all the HFD-fed groups (Table 3).

### 3.3. Effects of H. itama Bee Bread on Serum Glucose, Insulin Resistance and Lipid Profile

Indeed, hyperglycemia, hyperinsulinemia and insulin resistance are closely associated with obesity. To investigate the effects of bee bread on these parameters, the levels of glucose, insulin and HOMA-IR were evaluated in this current experiment. As shown in Table 4, the OB group showed significantly increased (*p* < 0.05) blood glucose and insulin levels after 12 weeks of HFD intervention compared to the CON group. In addition, compared with the CON group, the rats in the OB group also demonstrated a markedly higher (*p* < 0.05) insulin resistance index, HOMA-IR. However, bee bread supplementation significantly decreased (*p* < 0.05) blood glucose and insulin levels as well as improved the insulin resistance as shown by the reduced (*p* < 0.05) HOMA-IR index and those effects were also present in the OB + OR group, except for the HOMA-IR index. 

In addition, biochemical analysis of the lipid profile was also evaluated in the present study to determine the effect of bee bread on hyperlipidemia. After 12 weeks of HFD administration, compared to the CON group, serum TG, TC and LDL-C were increased significantly (*p* < 0.05), meanwhile serum HDL was reduced significantly (*p* < 0.05) in the OB group. Bee bread treatment significantly reduced (*p* < 0.05) these serum lipid levels, except for HDL-C, compared to those in the OB group, whereas, orlistat administration markedly reduced (*p* < 0.05) serum TC and LDL-C and significantly increased (*p* < 0.05) serum HDL-C compared to those in the OB group (Table 4). 

### 3.4. Effects of H. itama Bee Bread on Accumulations of Hepatic Lipid, NASH Activity and Glycogen

**Figure 2:**
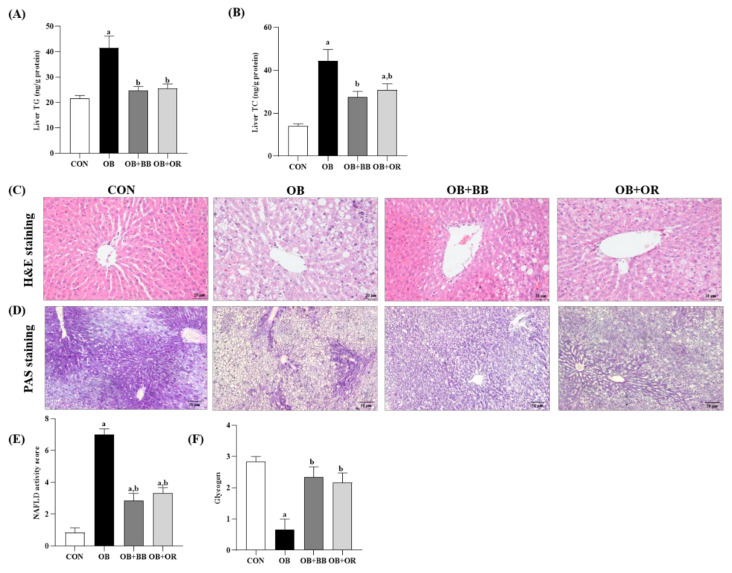
Effect of bee bread on liver lipid levels and hepatic steatosis. (**A**) Liver TG level, (**B**) liver TC level, (**C**) H&E staining (magnification ×400, scale bars represent 20 μm) and (**D**) PAS staining (magnification ×100, scale bars represent 50 μm) analyses of liver, (**E**) NASH scoring, and (**F**) glycogen scoring of rats in all experimental groups. Values are presented as mean ± SEM, *n* = 8/group. CON, control; OB, obese; OB + BB, obese + bee bread 0.5 g/kg body weight/day; OB + OR, obese + orlistat 10 mg/kg body weight/day; TG, triglyceride; TC, total cholesterol. One-way ANOVA, followed by Tukey post-hoc test. ^a^ *p* < 0.05 vs. CON group, ^b^ *p* < 0.05 vs. OB group.

The liver is a vital organ for controlling lipid metabolism and prolonged excessive lipid accumulation commonly leads to hepatic steatosis. Figure 2A,B demonstrates the significantly increased (*p* < 0.05) hepatic TG and TC levels in the OB group after 12 weeks of HFD intake, compared to the CON group, whereas, treatments with bee bread and orlistat markedly alleviated (*p* < 0.05) these hepatic lipid contents.

Photomicrographs of the liver samples stained with H&E are shown in Figure 2C. In the CON group, the liver structure was normal without any pathological symptoms. In this specimen, there was high preservation of hepatocytes and the lining cells of both sinusoids and postinusoidal venules, as well as the structural integrity of the hepatic lobule. In the hepatocytes of obese rats, a large number of lipid droplets (micro- and macro-vesicular steatosis) were observed. The liver tissue from this group also had more dilated sinusoids, portal triads and inflammatory cells infiltrated largely deposited at the portal region. In contrast, these histopathological changes were clearly reduced in the bee bread and orlistat groups although they were not completely reversed. Moreover, the NAS score of the liver samples is shown in Figure 2E. Clearly, the liver from the CON group had no presence of MAFLD. In contrast, the liver of the OB group demonstrated an average NAS of 7, which markedly demonstrated the presence of NASH in this group. Treatments with bee bread and orlistat demonstrated an average NAS of ≤4 which revealed the presence of simple steatosis in these groups. 

Increased hepatic gluconeogenesis, reduced glycogenesis and elevation in lipogenesis are common features of impaired hepatic insulin sensitivity. Therefore, we next evaluated the effect of bee bread on glycogen storage in the liver tissue using PAS staining (Figure 2D). The livers of the CON group displayed a high PAS staining intensity, which reflected the number of glycogen particles in the cytoplasm of hepatocytes, whereas, the staining intensity in the OB group was dramatically alleviated. Moreover, in the area of severe fat accumulation, positive staining was barely observed. The staining intensity of glycogen in the liver of the bee bread-treated group was much stronger than that in the OB group and this finding was similarly observed in the orlistat group. In addition, Figure 2F shows the grading for glycogen in all the liver sections. The results demonstrated a substantially decreased (*p* < 0.05) glycogen score in the OB group in comparison with the CON group, meanwhile, treatments with bee bread and orlistat significantly improved (*p* < 0.05) these changes.

### 3.5. Effect of H. itama Bee Bread on Liver Oxidant–Antioxidant Parameters

**Figure 3:**
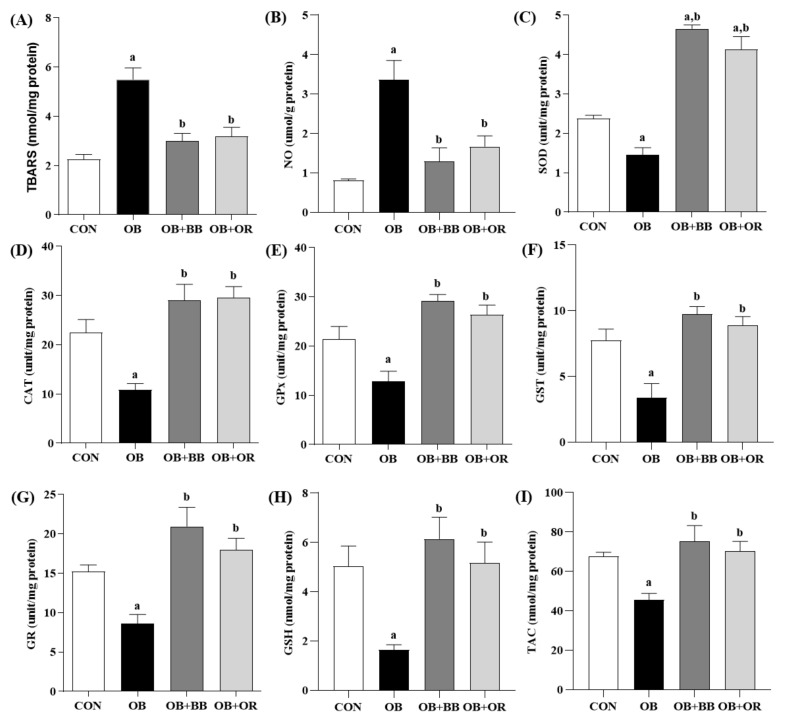
Effect of bee bread on liver oxidant–antioxidant parameters of rats in all experimental groups. (**A**) TBARS, (**B**) NO, (**C**) SOD, (**D**) CAT, (**E**) GPx, (**F**) GST, (**G**) GR, (**H**) GSH and (**I**) TAC in the liver of obese rats. Values are presented as mean ± SEM, *n* = 8/group. CON, control; OB, obese; OB + BB, obese + bee bread 0.5 g/kg body weight/day; OB + OR, obese + orlistat 10 mg/kg body weight/day; TBARS, thiobarbituric acid reactive substances; NO, nitric oxide; SOD; superoxide dismutase; CAT; catalase; GPx, glutathione peroxidase; GST, glutathione S-transferase; GR; glutathione reductase; GSH, total glutathione; TAC; total antioxidant capacity. One-way ANOVA, followed by Tukey post-hoc test. ^a^ *p* < 0.05 vs. CON group, ^b^ *p* < 0.05 vs. OB group.

**Figure 4:**
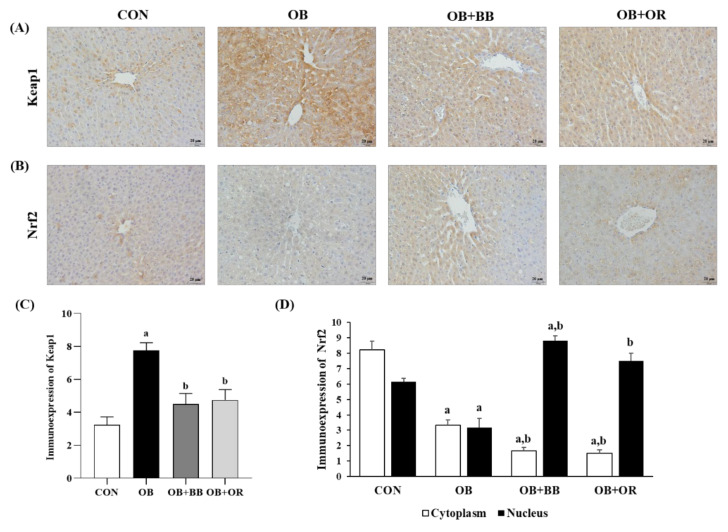
Effect of bee bread on (**A**) Keap1 and (**B**) Nrf2 immunohistochemical expressions in liver sections of rats in all experimental groups. (**C**) Immunohistochemical analyses of Keap1 and (**D**) cytoplasmic and nuclear Nrf2 in liver sections. Magnification ×400, scale bars represent 20 μm. Values are presented as mean ± SEM, *n* = 6/group. CON, control; OB, obese; OB + BB, obese + bee bread 0.5 g/kg body weight/day; OB + OR, obese + orlistat 10 mg/kg body weight/day; Keap1, Kelch-like ECH-associated protein 1; Nrf2, nuclear factor erythroid 2-related factor 2. One-way ANOVA, followed by Tukey post-hoc test. ^a^ *p* < 0.05 vs. CON group, ^b^ *p* < 0.05 vs. OB group.

Oxidative stress is linked with obesity-related fatty liver disease. We explored the effects of bee bread supplementation on several oxidant-antioxidant markers, as well as on the Keap1 and Nrf2 pathways as reported in Figure 3 and Figure 4, respectively. Our results showed that, compared to the CON group, there were significantly increased (*p* < 0.05) concentrations of oxidative stress markers including TBARS and NO in the OB group. Furthermore, there were also significant reductions (*p* < 0.05) in the activities of SOD, CAT, GPx, GST and GR enzymes, as well as in the levels of GSH and TAC in the OB group compared to the CON group. Treatment with bee bread notably reversed (*p* < 0.05) all these effects and equivalent outcomes were also demonstrated by the OB + OR group following orlistat treatment (Figure 3A–I).

Next, we used immunohistochemical analysis to further discuss the antioxidative effect of bee bread in activating the Keap1/Nrf2 pathway in the liver of obese rats, as shown in Figure 4. There was a significantly increased (*p* < 0.05) liver Keap1 expression level in the cytoplasm of obese rats, whereas, 6-week bee bread treatment significantly suppressed (*p* < 0.05) this Keap1 expression level (Figure 4A,C). Furthermore, the results also showed that, compared to the control group, the expressions of Nrf2 both in the cytoplasm and nucleus decreased (*p* < 0.05) after overfeeding of HFD for 12 weeks in the obese group, which indicated a lower rate of Nrf2 translocation from the cytoplasm into the nucleus. At the same time, the immunoexpression results clearly showed that the expression of cytoplasmic Nrf2 was significantly decreased (*p* < 0.05) and the expression of nuclear Nrf2 was significantly increased (*p* < 0.05) after bee bread treatment, and the results were also comparable in the orlistat group when compared to the obese group. These results demonstrated a higher translocation of cytoplasmic Nrf2 into the nucleus following bee bread treatment and these findings were similarly observed after orlistat administration in the OB + OR group (Figure 4B,D).

### 3.6. Effects of H. itama Bee Bread on the Expression of Hepatic Lipid Metabolism-Related Genes, and SIRT1 and AMPK Protein Levels

**Figure 5:**
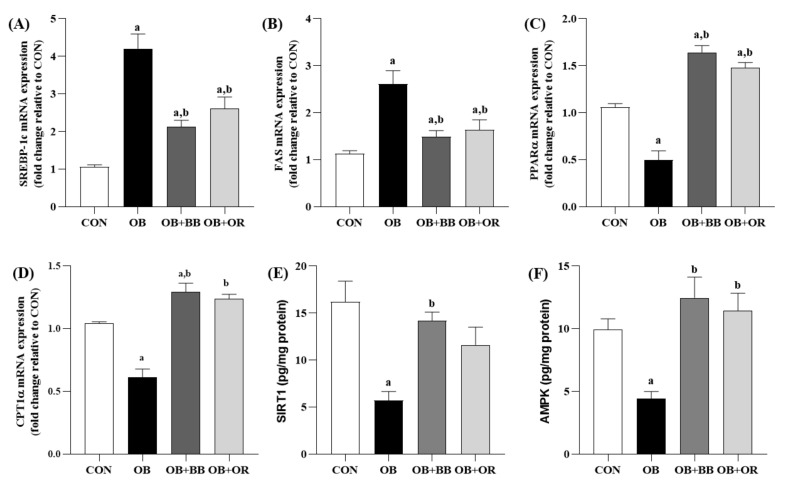
Effect of bee bread on hepatic lipid metabolism-related genes, and SIRT1 and AMPK levels. (**A**–**D**) The mRNA expression levels of SREBP-1c, FAS, CPT1α and SIRT1 were determined by RT-qPCR assays (*n* = 6/group). (**E**,**F**) The levels of SIRT1 and AMPK were detected using ELISA (*n* = 8/group). Values are presented as mean ± SEM. CON, control; OB, obese; OB + BB, obese + bee bread 0.5 g/kg body weight/day; OB + OR, obese + orlistat 10 mg/kg body weight/day; SREBP-1c, sterol regulatory element-binding protein-1c; FAS, fatty acid synthase; PPARα, peroxisome proliferator activated receptor α; CPT1α, carnitine palmitoyl transferase 1α; SIRT1, sirtuin 1; AMPK, AMP-activated protein kinase. One-way ANOVA, followed by Tukey post-hoc test. ^a^ *p* < 0.05 vs. CON group, ^b^ *p* < 0.05 vs. OB group.

To further investigate the underlying mechanisms by which bee bread improves hepatic lipid metabolism, we analyzed the effects of bee bread on genes related to lipogenesis and fatty acid β-oxidation as shown in Figure 5A–D. Compared with the CON group, the mRNA expression levels of both SREBP-1c and FAS were significantly up-regulated (*p* < 0.05) in the liver tissues of rats in the OB group. Treatment with bee bread for 6 weeks significantly inhibited lipid synthesis as shown by the down-regulation (*p* < 0.05) of these mRNA factors and the effects were also comparable after intake of orlistat. Furthermore, our results also demonstrated significantly down-regulated (*p* < 0.05) PPARα and CPT1α mRNA expression levels in the OB group than those in the CON group, whereas, these effects were significantly abolished (*p* < 0.05) by both bee bread and orlistat treatments.

The present study also evaluated the relationship between the anti-lipid effect of bee bread and the levels of SIRT1 and AMPK in the liver as demonstrated in Figure 5E,F). Our results showed that rats in the OB group had notably suppressed (*p* < 0.05) SIRT1 and AMPK levels more than those in the CON group. Meanwhile, bee bread treatment markedly elevated (*p* < 0.05) these protein levels. Comparable outcomes (*p* < 0.05) were also demonstrated in the liver of rats in the orlistat group except for the SIRT1 level which was not significantly changed (*p* > 0.05) after 6 weeks of treatment.

## 4. Discussion

Obesity is a chronic metabolic disease that can affect the majority of the vital organs of the body, and studies have reported that obesity is a high-risk factor for the development of cardiovascular diseases [68], type 2 diabetes [69] and carcinoma [70]. Apart from increases in the size of adipocytes (hypertrophy) and number of adipocytes (hyperplasia), being overweight and obese also leads to the excessive accumulation of lipid in the liver, resulting in hepatic steatosis, the early stage of MAFLD [71]. We had previously reported excessive intake of HFD results in elevated liver fat contents, oxidative damage and abnormal inflammatory responses in obese rat models fed with HFD for 12 weeks. Subsequently, the supplementation of *H. itama* bee bread, concurrently given with the HFD for the same period exerted its hepatoprotective effect against the above parameters [40]. In addition, the latest study by Li et al. 2021 [41] also demonstrated the anti-lipogenic effect of bee bread against FAS and ACC levels in the HFD-induced fatty liver disease rat model. Hence, to date it is not known whether *H. itama* bee bread has a therapeutic effect in treating MAFLD after the induction of obesity in the animal model.

In the present study, we found that *H. itama* bee bread is rich in phenols (2-hydroxycinnamic acid, trans 3-hydroxycinnamic acid, trans ferulic acids) and flavonoids (caffeic acid, apigenin, kaempferol, quercetin, mangiferin). The highest amount of compound found in both aqueous and methanol extracts of this bee bread was trans 3-hydroxycinnamic acid, followed by 2-hydroxycinnamic acid and quercetin. This finding is consistent with the Indian and Romanian bee bread samples which demonstrated a relatively high amount of hydroxycinnamic acid derivatives, quercetin and kaempferol [72]. However, it is suggested that the exact amount of each phenolic compound present in the *H. itama* bee bread is further quantitated in future studies. Both hydroxycinnamic acid derivatives and quercetin exhibit numerous biological and pharmacological effects, such as anti-obesity, anti-lipid, antioxidant and anti-inflammatory activities, and the potential therapeutic benefits in experimental diabetes and hyperlipidemia [73,74,75,76,77]. Our results suggest that *H. itama* bee bread may serve as the treatment for obesity-related metabolic disorders including hyperglycemia, insulin resistance and hyperlipidemia in MAFLD. We also focused on and demonstrated the therapeutic effects of *H. itama* bee bread in inhibiting Keap1, activating the Nrf2 antioxidant pathway and then restoring the dysregulation of genes associated with hepatic lipid metabolism such as SREBP-1c, FAS, PPARα and CPT1α, as well as its modulators such as SIRT1 and AMPK, being consistent with its increase in glycogen accumulation and reduction in hepatic steatosis and progression towards NASH in the liver of obese male rats.

The present study demonstrated significant increases in the Lee obesity index and body weight gain in the obese group than those in the control group, which are consistent with our previously published reports [40,78]. Furthermore, excess energy or calorie intake following consumption of HFD led to an increased accumulation of lipids in the adipocytes, hence resulting in an increased Lee obesity index and body weight gain in this group, as reported by previous studies [33,37]. *H. itama* bee bread was able to reduce these parameters significantly although no changes in food and calorie intakes were observed in this group, compared to the obese group. These results demonstrated the anti-obesity effect of *H. itama* bee bread in which it increased weight loss without reducing the food intake; hence, these results were in line with previous published studies [33,37,40].

Indeed, many studies have reported the presence of a strong relationship between obesity and impaired insulin sensitivity including a reduction in the number of insulin receptors and receptor function defects, leading to hyperglycemia, hyperinsulinemia and insulin resistance [16,79,80,81]. The present study demonstrated that long-term intake of an HFD significantly elevated the fasting blood glucose and insulin levels in the obese group than those in the control group, meanwhile *H. itama* bee bread supplementation markedly reduced these parameters. In addition, the HOMA-IR index was also used in the present study, which is a common indicator to evaluate insulin sensitivity, insulin resistance and pancreatic β-cell function in diabetic patients [82,83]. Our present research showed that the HOMA-IR index was significantly increased in the obese group than in the control group, meanwhile, the HOMA-IR index was significantly reduced in the bee bread group compared with the obese group. These results showed that *H. itama* bee bread treatment alleviated obesity-induced hyperglycemia, hyperinsulinemia and insulin resistance, hence improving insulin sensitivity [40].

Moreover, hyperlipidemia is also commonly presented in obese patients and recognized as one of the hallmarks of MAFLD [84,85]. Our findings presented significant increased TG, TC and LDL-C levels and a significantly reduced HDL-C level in the obese group than those in the control group, hence indicating the presence of hyperlipidemia in this group, which is consistent with other previously reported studies in *Sprague Dawley* rats fed with HFD [86,87]. The bee bread-treated group showed significantly reduced serum levels of non-HDL lipids without affecting the level of HDL-C in this group, which is in agreement with a prior finding by Othman et al. 2021 [34]. The present study, however, contradicts our previous study using *H. itama* bee bread on MAFLD’s rat protective model. The hypolipidemic effect of this bee bread might be attributed to the high amounts of phenols and flavonoids found in the *H. itama* bee bread sample as reported in a prior study [46]. Likewise, a former finding also reported that the potential of bee bread in reducing the non-HDL lipids might be through the action of saponin present in the bee bread which interacts with the dietary fat compositions and excretes out the lipids from the body via the feces [88].

Although the underlying mechanism for the development and progression of MAFLD is complex and multifactorial, it has been generally believed that MAFLD is a disease caused by a “second hit” with excessive lipid peroxidation and oxidative stress after hepatic fat accumulation (hepatic steatosis) that serves as the “first hit” [89]. Overfeeding of dietary fats causes the liver to become susceptible to toxins and oxidative stress, resulting in hepatocyte inflammation by the activation of oxidative stress, pro-inflammatory cytokines and hepatocyte mitochondrial dysfunction [90]. *H. itama* bee bread reduced TBARS and NO levels and increased the activities of SOD, CAT, GPx, GST and GR, as well as the levels of GSH and TAC in the liver of obese rats. These findings are correlated with previously reported studies using this bee bread which showed raised antioxidant enzyme activities in the liver [40], testis [39], aorta [35], heart [34] and renal [36] of the obese rats. The Nrf2 antioxidant pathway is a central cellular system in its defense against oxidative stress [91,92]. Suppression of Keap1 activity reduces the production of ROS, meanwhile Nrf2 knockout leads to an increased accumulation of ROS in mouse primary hepatocytes [93]. Elevated lipid peroxidation resulting from excess lipid accumulation in the liver promotes the dissociation of Nrf2 from Keap1 and translocation of Nrf2 from the cytoplasm into the nucleus to activate the antioxidant pathway by binding to the ARE in the promoter region of antioxidant and stimulates the expressions of antioxidant enzymes [94]. Recent studies have reported that HFD stimulates liver oxidative stress via the inhibition of Nrf2 nuclear translocation to suppress the expression of antioxidant enzymes [15,95]. Our present study demonstrated the up-regulated expression of Keap1 and the reduced translocation of Nrf2 from the cytoplasm into the nucleus in the liver of rats from the obese group. Meanwhile, *H. itama* bee bread was found to inhibit Keap1 and promote the translocation of Nrf2 from the cytoplasm into the nucleus. These results were in parallel with the increased activities of antioxidant enzymes such as SOD, CAT, GPx, GST and GR in the liver of the bee bread group. The increased activities of antioxidant enzymes could be due to increased expressions of antioxidant genes, the measurements of which should be undertaken in future studies. Keap1 suppression and Nrf2 antioxidant pathway activation with the high activities of antioxidant enzymes by *H. itama* bee bread might mitigate HFD-triggered ROS, subsequently effectively restoring hepatic redox status imbalance in the obese rats. The beneficial property of *H. itama* bee bread demonstrated in the present study might be attributed to its rich sources of phenolic compounds such as hydroxycinnamic acid derivatives, caffeic acid, gallic acid, ferulic acid, quercetin, apigenin, kaempferol and mangiferin, which are reported to possess antioxidant properties [44,75,96].

The imbalance between lipid acquisition (i.e., high fatty acid uptake and lipogenesis) and removal (i.e., low mitochondrial fatty acid oxidation and export of lipids) can result in an impaired lipid metabolism which later leads to several metabolic disorders including obesity, metabolic syndromes and MAFLD [97]. Of note, Nrf2 has been shown to inhibit ethanol-induced hepatic steatosis in mice [93,98]. Meanwhile, Keap1-knockout with Nrf2-enhanced mice suppressed ethanol-induced hepatic fat accumulation by reducing the expressions of SREBP-1 and SCD-1 genes in mice [93]. Similarly, Nrf2 deficiency down-regulates PPARα but increases SREBP-1c to enhance TG contents in the ethanol-exposed human hepatocytes cell line, whereas, increased Nrf2 expression reversed these malfunctions [98]. In our study, hyperinsulinemia with increased gene expression of SREBP-1c and its target gene FAS were found in the obese group compared to the control group. *H. itama* bee bread was shown to down-regulate these gene expression levels which might be accredited to reduced hyperinsulinemia in the bee bread-treated group. These findings are in line with a previous study using bee bread in HFD-induced fatty liver rats [41]. In addition, the decreased lipogenesis in this group following *H. itama* bee bread administration might explain the reduced contents of hepatic TG and TC in this group, which is in line with previous studies [16,99]. Our results demonstrated an impairment in hepatic fatty acid β-oxidation in the obese group, as indicated by the reductions in PPARα and CPT1α mRNA levels in this group. These results are in agreement with previous findings which reported that the disruption in fatty acid β-oxidation is associated with reductions in PPARα and CPT1α expressions or activities in humans and rodents with MAFLD [100,101,102,103]. Our study demonstrated that *H. itama* bee bread can enhance PPARα and CPT1α mRNA expression levels, hence increasing the fatty acid β-oxidation in the obese rats treated with bee bread. Furthermore, the reduced liver TG and TC contents in the bee bread group might also be attributed to the therapeutic ability of *H. itama* bee bread in increasing fatty acid β-oxidation and ATP production, as well as to reduce inflammation and oxidative stress [40]. Therefore, the down-regulation of *H. itama* bee bread on oxidative stress via the mediation of the Keap1/Nrf2 pathway might play a role in reducing liver lipid deposition in obese rats.

The liver of obese rats has reduced AMPK activation [104,105,106]. SIRT1 activates AMPK expression, promotes AMPK phosphorylation and suppresses SREBP-1c cleavage and nuclear translocation [20]. Polyphenols including mangiferin, resveratrol, quercetin and catechin, which were also found in the *H. itama* bee bread of the present study, have been reported to be one of the SIRT1 inducers and improve hepatic lipid metabolism via the promotion of the SIRT1/AMPK pathway in vitro and in vivo of the MAFLD model [99,107,108]. Moreover, treatment with bee pollen polysaccharide from *Rosa rugosa* alleviated hepatic steatosis and insulin resistance by promoting the phosphorylation of AMPK in HepG2 cells and HFD-induced animal models [109]. Previous findings have demonstrated that excessive lipid deposition in the liver can restrain the AMPK substrate ACC phosphorylation, promoting lipid synthesis [71]. A reduced level of AMPK may result in excessive hepatic lipid accumulation, accelerating steatosis and MAFLD, hence further demonstrating that AMPK is an energy sensor regulator to maintain lipid and glucose metabolism in the liver. It is also has been reported that the activation of AMPK promotes the Nrf2 antioxidant pathway [110,111]. In this study, we demonstrated that *H. itama* bee bread treatment significantly elevated SIRT1 and AMPK levels and the results were consistent with the reduced insulin resistance levels of lipogenic genes SREBP-1c and FAS, as well as levels of TG and TC in the liver, which might be accredited to its activation on Keap1/Nrf2 signaling pathway. However, it is suggested that the level of activated AMPK (p-AMPK) is measured in future studies to further support these findings.

Prolonged intake of HFD leads the liver to store more TGs, causing hepatic steatosis and stimulating the development of NASH, which can further progress to fibrosis and cirrhosis [112]. In the present study, the histopathological finding using H&E staining also revealed that the liver of rats in the obese group demonstrated pronounced hepatic steatosis, hepatocyte hypertrophy and hepatocellular inflammation, resulting in an elevated NAS score, hence indicating the presence of active NASH in this group. *H. itama* bee bread treatment mitigated hepatic steatosis and inflammation, significantly reducing the NAS score and preventing NASH in comparison with the rats in the obese group. These results further support our previous reported data on the beneficial protective effect of *H. itama* bee bread against NASH progression in the HFD-induced obese rats [40]. In addition, peripheral and hepatic insulin resistance, which are linked to the development of MAFLD, lead to insufficient suppression of hepatic gluconeogenesis, reduced glycogenesis and elevation in lipogenesis [113]. In the present study, we found that *H. itama* bee bread was able to significantly recover the reduced glycogen accumulation in the liver tissue of obese rats which might indicate that this bee bread could modulate hepatic glycogenesis, increase insulin sensitivity and maintain the metabolic function of the liver.

## 5. Conclusions

We found that treatment with *H. itama* bee bread inhibited Keap1 and activated the Nrf2 antioxidant pathway with a reduction in oxidative stress in obese rat livers. This therapeutic potential of *H. itama* bee bread subsequently improved lipid metabolism-related genes (SREBP-1c, FAS, PPARα and CPT1α) and its regulators SIRT1 and AMPK; hence, lipid accumulation and the progression of NASH were alleviated in the liver of obese rats. These improvements might be partly attributed to the presence of phenolic compounds in the *H. itama* bee bread which activate the Keap1/Nrf2 signaling pathway.

## Figures and Tables

**Table 1:** Primers used for PCR amplification.

Gene Name	Accession Number	Sequence	Reference
SREBP-1c	NM_001276707.1	Forward: 5′-GCCTGCTTGGCTCTTCTCT-3′ Reverse: 5′-GCTTGTTTGCGATGTCTCC-3′	[62]
FAS	NM_017332.1	Forward: 5′-TCGACTTCAAAGGACCCAGC-3′ Reverse: 5′-ACTGCACAGAGGTGTTAGGC-3′	[63]
PPARα	NM_013196.1	Forward: 5′-ATTCGGCTAAAGCTGGCGTA-3′ Reverse: 5′-TGCATTGTGTGACATCCCGA-3′	[63]
CPT1α	BC072522.1	Forward: 5′-GGACATTCCTCTCTCAGGTTTC-3′ Reverse: 5′-ACCTCCTCCTTTGAACACATAC-3′	[63]
GAPDH	NM_017008	Forward: 5′-TCACCACCATGGAGAAGGC-3′ Reverse: 5′-GCTAAGCAGTTGGTGGTGCA-3′	[39]

SREBP-1c, sterol regulatory element-binding protein-1c; FAS, fatty acid synthase; PPARα, peroxisome proliferator activated receptor α; CPT1α, carnitine palmitoyl transferase 1α; GAPDH, glyceraldehyde 3-phosphate dehydrogenase.

**Table 2:** Contents of phenolic compounds in the aqueous and methanol extracts of bee bread.

Phenolic Compound	Molecular Formula	Retention Time (min)	Area (mAU × min)	Relative Area (%)
Aqueous extract:				
Gallic acid	C_7_H_6_O_5_	5.93	1.106	1.59
Mangiferin	C_19_H_18_O_11_	13.09	1.093	1.57
Trans 3-hydroxycinnamic acid	C_9_H_8_O_3_	15.05	40.235	57.77
2-hydroxycinnamic acid	C_9_H_8_O_3_	15.77	1.379	1.98
Methanol extract:				
Caffeic acid	C_9_H_8_O_4_	12.53	2.153	0.50
Mangiferin	C_19_H_18_O_11_	13.09	4.170	0.97
Trans ferulic acid	C_10_H_10_O_4_	14.62	1.035	0.24
Trans 3-hydroxycinnamic acid	C_9_H_8_O_3_	15.05	74.221	17.31
2-hydroxycinnamic acid	C_9_H_8_O_3_	15.77	4.909	1.15
Quercetin	C_15_H_10_O_7_	17.64	18.878	4.40
Kaempferol	C_15_H_10_O_6_	18.73	7.999	1.87
Apigenin	C_15_H_10_O_5_	19.01	9.499	2.22

**Table 3:** Obesity parameters and nutritional composition of rats in the experimental groups.

	CON	OB	OB + BB	OB + OR
Lee obesity index	306.4 ± 1.60	331.5 ± 2.69 ^a^	311.4 ± 2.45 ^b^	316.9 ± 2.64 ^a,b^
Body weight gain (g)	103.0 ± 9.26	204.7 ± 10.98 ^a^	154.9 ± 14.47 ^a,b^	157.0 ± 6.98 ^a,b^
Average daily food intake (g/day)	20.97 ± 0.50	20.65 ± 0.71	18.58 ± 0.70	19.08 ± 0.72
Average daily calorie intake (kJ/day)	282.7 ± 6.47	446.2 ± 15.35 ^a^	401.5 ± 15.17 ^a^	412.3 ± 15.46 ^a^

Data are presented as mean ± SEM, *n* = 8/group. CON, control; OB, obese; OB + BB, obese + bee bread 0.5 g/kg body weight/day; OB + OR, obese + orlistat 10 mg/kg body weight/day. One-way ANOVA, followed by Tukey post-hoc test. ^a^ *p* < 0.05 vs. CON group, ^b^ *p* < 0.05 vs. OB group.

**Table 4:** Serum fasting glucose, insulin resistance and lipid profile of rats in the experimental groups.

	**CON**	**OB**	**OB + BB**	**OB + OR**
Fasting glucose (mg/dL)	70.50 ± 1.45	83.00 ± 3.54 ^a^	73.22 ± 1.36 ^b^	74.50 ± 1.93 ^b^
Fasting insulin (ng/mL)	0.64 ± 0.09	3.71 ± 1.33 ^a^	1.22 ± 0.22 ^b^	1.80 ±0.09 ^b^
HOMA-IR	0.12 ± 0.01	0.38 ± 0.07 ^a^	0.22 ± 0.03 ^b^	0.26 ± 0.05 ^a^
TG (mmol/L)	0.49 ± 0.03	0.92 ± 0.03 ^a^	0.60 ± 0.06 ^b^	0.77 ± 0.08 ^a^
TC (mmol/L)	1.66 ± 0.10	2.79 ± 0.40 ^a^	1.97 ± 0.06 ^b^	2.01 ± 0.09 ^b^
LDL-C (mmol/L)	0.80 ± 0.04	1.80 ± 0.24 ^a^	1.12 ± 0.10 ^b^	1.03 ± 0.08 ^b^
HDL-C (mmol/L)	0.53 ± 0.02	0.40 ± 0.02 ^a^	0.49 ± 0.04	0.66 ± 0.03 ^a,b,c^

Data are presented as mean ± SEM, *n* = 8/group. CON, control; OB, obese; OB + BB, obese + bee bread 0.5 g/kg body weight/day; OB + OR, obese + orlistat 10 mg/kg body weight/day; HOMA-IR, homeostatic model of assessment-insulin resistance; TG, triglyceride; TC, total cholesterol; LDL-C, low-density lipoprotein cholesterol; HDL-C, high-density lipoprotein cholesterol. One-way ANOVA, followed by Tukey post-hoc test. ^a^ *p* < 0.05 vs. CON group, ^b^ *p* < 0.05 vs. OB group, ^c^ *p* < 0.05 vs. BB group.

## Data Availability

All of the data are contained within the article.

## Supplementary Materials

The following are available online at https://www.mdpi.com/article/10.3390/antiox11112190/s1, Table S1: Lee obesity index and liver enzymes, Figure S1: Histology of liver.
