# Supplementary material for: Therapeutic Effects of Heterotrigona itama (Stingless Bee) Bee Bread in Improving Hepatic Lipid Metabolism through the Activation of the Keap1/Nrf2 Signaling Pathway in an Obese Rat Model
Source: Antioxidants (Basel). 2022 Nov 5;11(11):2190. doi: 10.3390/antiox11112190 (PMC9686663; doi:10.3390/antiox11112190)
Supplement: Supplementary file 1 [file antioxidants-11-02190-s001.zip › antioxidants-1972467-supplementary.pdf]

Pilot study:

Table S1 Lee obesity index and liver enzymes.

Parameters	N	HFD	HFD+0.5Bb	HFD+1.0Bb	HFD+1.5Bb
Lee obesity index	299.0 ± 12.03	318.3 ± 10.93	305.7 ± 7.23	316.7 ± 16.26	318.0 ± 6.35
Liver enzymes:					
ALP	192.33 ± 34.98	597.67 ± 166.04	301.00 ± 29.45	353.00 ± 55.30	446.67 ± 148.62
AST	83.33 ± 5.47	156.33 ± 30.75	142.67 ± 18.27	153.33 ± 25.43	143.00 ± 61.27
ALT	42.33 ± 2.07	93.00 ± 25.94	83.33 ± 9.31	126.67 ± 32.46	138.33 ± 95.06

Data are presented as mean ± SD, $n = 3$ per group. N, Normal; HFD, high-fat diet; HFD+0.5Bb, HFD plus 0.5 g/kg body weight/day bee bread; HFD+1.0Bb, HFD plus 1.0 g/kg body weight/day bee bread; HFD+1.5Bb, HFD plus 1.5 g/kg body weight/day bee bread; ALP; alanine phosphatase; AST; aspartate transferase; ALT; alanine transferase. No significant differences were found between the groups (One-way ANOVA). However, the Lee obesity index was the highest in the HFD group, followed by HFD+1.5Bb, HFD+1.0Bb, HFD+0.5Bb and N groups. Similar trends of results as the Lee obesity index were also observed in the level of ALP in all groups. The level of AST enzyme was the highest in the HFD group, followed by HFD+1.0Bb, HFD+1.5Bb, HFD+0.5Bb and N groups. Whereas, the level of ALT was the highest in the HFD+1.5Bb, followed by HFD+1.0Bb, HFD, HFD+0.5Bb and N groups.

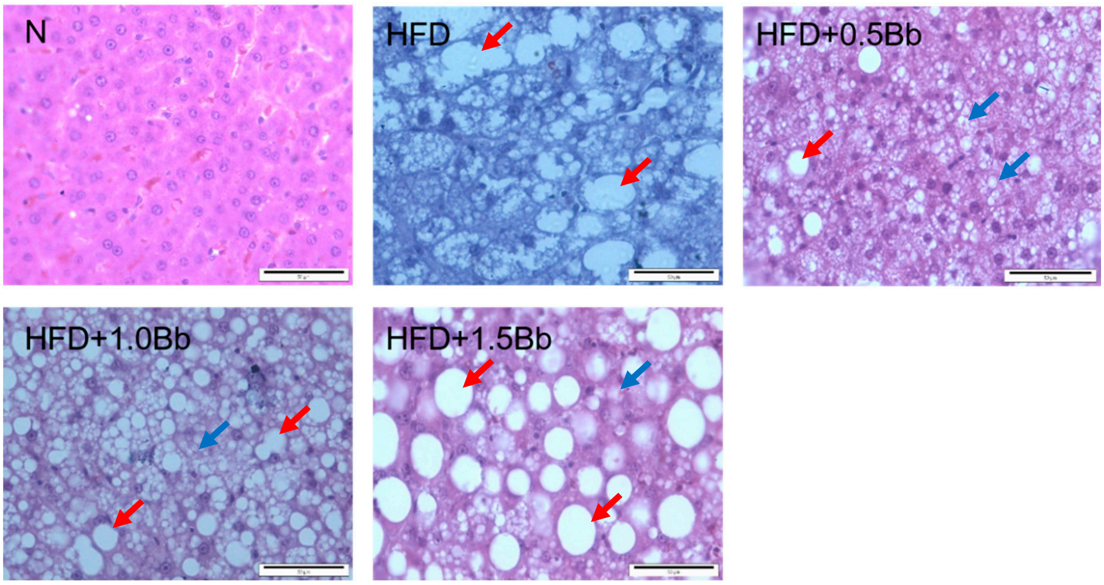

Figure S1 Histology of liver. N group shows normal histology of the liver. HFD group shows a high degree of fatty liver with severe cytoplasmic vacuoles and swelling of hepatocytes (red arrows). HFD+0.5Bb group, however, shows smaller number and size of cytoplasmic vacuoles and hepatocytes (blue arrows) compared to HFD, HFD+1.0Bb and HFD+1.5Bb groups. N, Normal; HFD, high-fat diet; HFD+0.5Bb, HFD+0.5 g/kg body weight/day bee bread; HFD+1.5Bb, HFD+1.5 g/kg body weight/day bee bread. (Haematoxylin and Eosin staining, magnification ×400, scale bar=50µm).
